# Extramedullary Manifestations of Chronic Myelomonocytic Leukemia: Do We Treat like an Acute Myeloid Leukemia?

**DOI:** 10.1155/2019/8360454

**Published:** 2019-11-30

**Authors:** Prasun Pudasainee, Bimatshu Pyakuryal, Yogesh Subedi, Jenisha Upadhyay, Subarna Adhikari

**Affiliations:** ^1^Department of Internal Medicine, Nepal Medical College, Kathmandu, Nepal; ^2^Department of Internal Medicine, Institute of Medicine, Kathmandu, Nepal; ^3^Mountain Medicine Society of Nepal, Kathmandu, Nepal

## Abstract

Chronic myelomonocytic leukemia (CMML) is a relatively rare clonal hematologic disorder with features of myelodysplastic syndrome and myeloproliferative disease. Extramedullary leukemic involvement is rarely a presenting feature of CMML. As there are no clear guidelines in regard to the treatment of patients with extramedullary manifestations, its management is challenging. In this report, we discuss the management of our patient who presented with submandibular lymphadenopathy and gingivitis and was diagnosed with CMML.

## 1. Introduction

CMML is a malignant hematopoietic stem cell disorder with clinical and pathological features of both myelodysplastic syndrome and myeloproliferative neoplasms (MDS/MPN). The median age at diagnosis of CMML is 71 years and has a median survival of 14–22 months [1]. Extramedullary manifestations of CMML are uncommon and are seen in organs such as spleen, liver, skin, and lymph nodes. Here, we present a case of CMML with extramedullary manifestations of myeloid sarcoma (MS) of the lymph node and gingival infiltration.

## 2. Case Presentation

**Figure 1:**
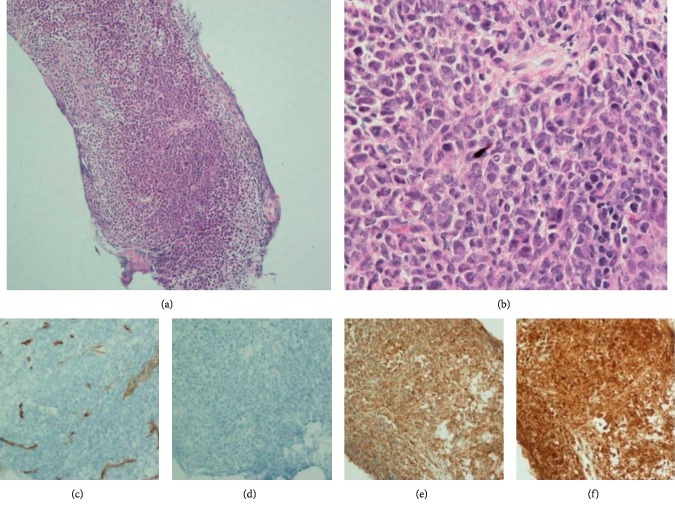
(a) Core needle biopsy of right submandibular mass. (b) Myeloid blast cells. (c) CD34 (+) blastic cells. (d) CD117 (+) blastic cells. (e) CD68 (+) blastic cells. (f) MPO (+) blastic cells.

A 40-year-old male with no significant past medical history presented to his primary physician with swelling on the right side of his neck and gum bleeding for a few weeks. He complained of fatigue, night sweats, and weight loss (∼25 lb) in the preceding two months. On examination, he had significant gum hypertrophy and mobile, nontender mass in the right submandibular area and right cheek. Lab results were significant for white blood cell count of 31,000/*μ*L (4% neutrophils, 74% monocytes, and 0.5% immature granulocytes). Bone marrow biopsy revealed hypercellular bone marrow (95%) with MDS/MPN features, consistent with CMML-1. Cytogenetics showed 48XY, +6, del(11)(q23), +19(16)/50∼53, idem, +4, +18[cp4]. JAK2 V617F mutation was negative. Fluorescence in situ hybridization (FISH) studies were normal and negative for platelet-derived growth factor receptor beta rearrangement PDGFRB (CEN)/PDGFRB (TEL). Ultrasound-guided biopsy of right submandibular mass and fine-needle aspiration of right cheek mass were consistent with myeloid sarcoma (Figure 1).

**Figure 2:**
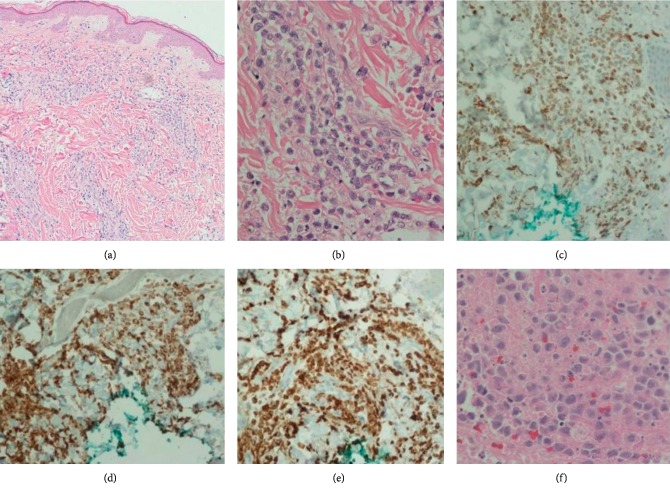
(a) Punch biopsy of the skin rash. (b) Atypical monocytes consistent with chronic myelomonocytic leukemia. (c) CD68 (+) monocytes. (d) Lysosome (+) monocytes. (e) MPO (+) monocytes. (f) Pleural fluid aspirate with atypical hematopoietic cells.

After discussing in the multidisciplinary tumor board, it was decided to treat this case as acute myeloid leukemia (AML). Induction with idarubicin and cytarabine on 3 + 7 protocol was performed. A repeat bone marrow biopsy was hypercellular without evidence of CMML; cytogenetics and FISH were normal. The patient subsequently proceeded with matched related allogenic hematopoietic cell transplant (HCT). Conditioning regimen included targeted busulfan and fludarabine. Graft-versus-host disease prophylaxis included tacrolimus and mycophenolate mofetil. The patient tolerated HCT fairly well, with only grade three mucositis and CMV reactivation. After five months of HCT, the patient developed skin rashes on the chest and right side of the abdomen. The biopsy of the skin was consistent with CMML (Figure 2).

Tacrolimus was discontinued. He received high-dose cytarabine salvage therapy followed by first donor lymphocyte infusion (DLI) at a 1 × 10^7^ kg cell dose. Since he had side effects with cytarabine, he received 10 days of decitabine with the second dose of escalated DLI (8 × 10^7^ kg cell dose). His second dose of DLI was given after five days of decitabine. However, the disease progressed with increased lymphadenopathy and new bony lesions. Despite three doses of DLI with decitabine, he developed large pleural effusion; analysis showed atypical hematopoietic cell population. Subsequently, he opted for hospice and died a week later.

## 3. Discussion

Myeloid sarcoma and gingival infiltration, although rare and described mostly with acute myeloid leukemia (AML), can be associated with CMML [2]. They may be the first manifestation of CMML, precede it by months or years, or represent the initial manifestation of relapse in a previously treated patient with CMML in remission. In our case, the patient presented with myeloid sarcoma and gingival infiltration at the time of CMML diagnosis, and subsequently had malignant pleural effusion with the progression of the disease. Myeloid sarcoma commonly involves skin, soft tissue, lymph nodes, bone, and periosteum [3]. Skin manifestations of AML can be nonspecific and may include pallor, petechiae or ecchymoses, neutrophilic dermatitis (Sweet syndrome), and infiltrative lesions such as leukemia cutis or myeloid sarcoma. Malignancy-associated Sweet's syndrome may occur in one of the three settings, either as paraneoplastic syndrome or drug-induced dermatosis or concurrently with leukemia cutis [4]. Pleural effusion secondary to malignant cell infiltration, similar to our case, has been reported rarely in AML [5]. CMML with extramedullary manifestations has a worse prognosis and needs aggressive treatment [6]. However, there are no randomized trials addressing the optimal management of CMML with extramedullary manifestations, and the data are limited to case reports and reviews. Thus, treatment is usually at clinicians' discretion in these cases. Importantly, management depends on whether extramedullary manifestations are regarded as the progression of CMML or transformation into acute leukemia. If considered as a progression of CMML, the patient is initially treated with hypomethylating agents. HCT, which is the only potential cure, is attempted after remission [7]. However, if CMML with extramedullary manifestations is considered AML-equivalent, different management with AML-type chemotherapy followed by HCT is used [3]. In our patient, we decided to pursue AML-type treatment based on age, aggressive presentation, and multiple extramedullary manifestations.

## 4. Conclusions

Extramedullary manifestations with CMML have a poor prognosis. Management should be individualized and depending on the presentation it can either be treated as AML or progression of CMML.
